# Volatile Organic Compounds Arising from Wood Polymers on Thermal Loading of Spruce Wood

**DOI:** 10.3390/polym17070875

**Published:** 2025-03-25

**Authors:** Katarína Trojanová, Veronika Veľková, František Kačík

**Affiliations:** 1 Department of Chemistry and Chemical Technology, Faculty of Wood Sciences and Technology, Technical University in Zvolen, 96001 Zvolen, Slovakia; xtrojanova@is.tuzvo.sk; 2 Department of Fire Protection, Faculty of Wood Sciences and Technology, Technical University in Zvolen, 96001 Zvolen, Slovakia; velkova@is.tuzvo.sk

**Keywords:** spruce wood, thermal treatment, flame retardant, emissions, gas chromatography–mass spectrometry (GC-MS)

## Abstract

The thermal degradation of wood polymers (cellulose, hemicelluloses, and lignin) results in the production of volatile products, some of which are toxic or act as irritants. In the present work, we focus on the effect of wood treatment on the formation of volatile products, conducting experiments on thermally treated (TTW), flame-retardant-treated (FRW), and untreated (REF) spruce wood. The samples were subjected to thermal loading at 150 °C, 200 °C, and 250 °C with the subsequent collection of degradation products. We evaluated the effect of wood treatment on the formation of volatile organic compounds (VOCs) using gas chromatography–mass spectrometry (GC-MS). The number and quantity of VOCs are significantly affected by the type of wood treatment and the thermal loading temperature. At the temperature of 250 °C, the concentration and number of VOCs increased significantly. The highest number of VOCs was identified in the untreated wood samples (54 compounds, mostly aldehydes, ketones, and phenols), with a lower number being identified in the flame-retardant-treated samples (9 compounds, mainly furfural) and the lowest number being identified in thermally treated wood samples (3 compounds, aliphatic hydrocarbons). Typical volatile products included furfural, furfurylalcohol, and α-pinene. Qualitative and quantitative analysis of VOCs under thermal loading is important in evaluating the wood burning process and the toxic properties of the consequent gaseous products.

## 1. Introduction

Wood is defined as the lignocellulosic substance between the pith and bark of a tree or a shrub. It is a renewable and readily processed material that has been widely used around the globe for a number of years. Chemically, wood primarily consists of three basic polymers, two of which are polysaccharides: linear, mostly crystalline cellulose (37–57%) and generally branched and amorphous hemicelluloses (16–37%). The third polymer is aromatic amorphous lignin (17–36%). Although wood is essentially polymeric, its structure and composition vary greatly between and within species [1,2].

Spruce wood is commonly used in the construction sector as a construction material, in pallets/packaging and fencing, and in the production of paper and panel products. When wood is heated, its structure changes depending on its original composition. Upon heating, the three polymeric components of wood begin to thermally decompose into a mixture of volatile gases, tar, and carbonaceous char. Decomposition is generally considered to be a superposition of the decomposition mechanisms of the individual components: hemicelluloses decompose first (180–350 °C), followed by cellulose (275–350 °C) and lignin (250–500 °C) [1]. It should be noted that lignin decomposes over a wide temperature range, depending on the degree of cross-linking and molecular weight distribution. Under thermal stress, wood polymers break down into shorter chains and then into monomers, which undergo numerous subsequent reactions to form a number of different compounds. The main products produced following the pyrolysis of wood are furfuryl alcohol, furfural, acetic acid, and levoglucosan from polysaccharides and eugenol and isoeugenol, vanillin, and guaiacols from the lignin [3,4].

Wood exhibits several implicit advantages compared with other materials (e.g., concrete, metals, and plastics); it is a permanently renewable source and has esthetically pleasing product qualities. Despite these advantages, wood also possesses unfavorable characteristics, such as weathering, flammability, and biodegradability [5]. Various treatment strategies are employed to extend the longevity of wood and protect it from fire. Thermal treatment of wood can improve the appearance of the wood product’s surface, its dimensional stability, and its resistance to fungal attacks [6]. Environmentally friendly techniques include thermal modification, a technique that does not involve the use of chemicals, with wood treated in this way exhibiting significantly increased stability and durability. Flame retardants are used to provide wood with protection against fire, and effective, environmentally friendly formulations are being researched in this regard [7,8,9].

During thermal modification processes, a certain amount of volatile organic compounds (VOCs), such as terpenes, aldehydes, acids, and alcohols, are released due to the thermal degradation of wood. The emission of organic waste gases containing high concentrations of VOCs can lead to severe atmospheric pollution, adversely affecting human health [10,11,12].

Thermal degradation is a process that depends on various factors, such as the environment in which the wood is located, the temperature of thermal loading, and the chemical composition of the wood itself. The composition of wood varies not only with the type of wood but also with the part of the wood that undergoes thermal degradation. In general, hemicelluloses are the first substances to degrade, together with lignin. At a temperature of around 200 °C, hemicelluloses decompose to form acetic acid, which also impacts the degradation of cellulose—it acts as a catalyst. Levoglucosan is formed from hemicelluloses and cellulose at high temperatures, forming flammable gases [13,14]. The thermal degradation process can be divided into several phases depending on the temperature. At temperatures of 50–100 °C, degradation processes associated with the formation of water and low-molecular-weight substances take place. At temperatures of 100–200 °C, a charred layer, formic acid, acetic acid, carbon monoxide, carbon dioxide, and others are formed. At temperatures between 200 °C and 300 °C, dehydration reactions are accelerated, and carbonyl, carboxyl, and hydroperoxide groups are formed. At temperatures between 300 °C and 450 °C, cellulose depolymerization and tar formation occur, and at temperatures above 450 °C, wood is oxidized to carbon monoxide and carbon dioxide and water [15,16,17].

The production of smoke and hazardous gases can threaten human health and safety, causing respiratory distress, nausea, vomiting, and even death. The primary products of smoke produced during the pyrolysis of wood are carbon monoxide (CO), carbon dioxide (CO_2_), and water (H_2_O). It should be noted, however, that the different cellulose and lignin contents of wood result in different product emissions. Cellulose decomposition at high temperatures generally results in the production of CO and hydrogen gas (H_2_); in comparison, lignin decomposition results in the production of complex phenolic aromatic products. The main products produced after the pyrolysis of wood are furfuryl alcohol, furfural, and levoglucosan from cellulose and eugenol and isoeugenol, vanillin, and guaiacols from lignin [1,18,19]. As the temperature increases, the decomposition of individual wood components produces both flammable and non-flammable gases, which, together with other factors (the oxygen concentration, char layer, and presence of a retardant), significantly impact the combustion process; moreover, some retardants (e.g., those containing nitrogen and halogens) can also produce toxic compounds [4,20,21]. Moisture content has a significant effect on the initiation of both flaming and smoldering combustion, as moisture can suppress the thermal pyrolysis of combustible gases during ignition and the evaporation of residual water can cause localized cooling of the surface after ignition. In addition, in one study, samples with high moisture content transitioned more rapidly and directly to smoldering combustion [22]. Wood is a complex multi-component biopolymer that exhibits significant variability, which is also reflected in its VOC emissions. Variability in VOC emissions or emission rates has been observed and is attributed to numerous endogenous and exogenous factors, including wood species, wood treatment, the heating method used, the environment, and the method of VOC sampling and analysis.

When analyzing VOC emissions from wood, these factors must therefore be considered to reduce their influence on measurements. It is recommended to describe them in such detail so as to facilitate a reliable interpretation of the results and to ensure comparability [23]. In addition, the conditions of gas sampling (temperature, time, sorption, and desorption method) also play an important role in the assessment of VOCs, with only the analytical method employed (gas chromatography–mass spectrometry, GC-MS) being almost uniform.

In light of the above findings, analysis of the products formed during the thermal loading of treated and untreated wood is important from both a fire prevention and health perspective. To assess the impact of thermal treatment and the use of flame retardants on the quality of VOCs compared to untreated spruce wood, experiments were carried out under the same conditions in the present study, providing a new perspective on the above factors. We aimed to determine the effect of wood thermal and flame-retardant treatment on the formation of volatile products at different temperatures of thermal loading.

## 2. Materials and Methods

Spruce wood (*Picea abies*) samples with dimensions of 20 mm × 20 mm × 20 mm were used for the experiments described herein. The first group of samples (TTW—thermally treated wood) was thermally modified at 160 °C for 9 h. The second group of samples (FRW—flame-retardant-treated wood) was treated with three coats of HR PROF (aqueous solution of iron orthophosphate (7–15%), citric acid, and special additives) retardant (Holz Prof OÜ, Tallinn, Estonia), which we applied using a brush at one-hour intervals. The total amount of used retardant was 300 g·m^−2^. Once all of the coats were applied, the samples were left to dry until they reached a moisture content of ca. 12%. The third group was left untreated and served as a reference sample (REF—reference wood).

The samples were thermally loaded in a quartz apparatus at temperatures of 150 °C, 200 °C, and 250 °C for 15 min, and the resulting volatile organic compounds (VOCs) were simultaneously collected in ANASORB CSC sampling tubes (SKC Ltd., Blandford Forum, UK) with activated carbon using an air sampling pump. The gas flow rate was adjusted to 1 L∙min^−1^. After heat treatment, the adsorbed volatiles were extracted with 3 mL of carbon disulfide for 20 min on a shaker. Subsequently, 1 mL of the extract was transferred to vials and analyzed using gas chromatography–mass spectrometry (GC-MS) under the following conditions: Agilent 7890B gas chromatograph (Agilent Technology, Palo Alto, CA, USA); column: HP5-MS; 30 m; 0.25 mm; 0.25 μm; carrier gas: He; carrier gas flow: 1 mL·min^−1^; temperature program: 40 °C for 4 min, increase 5 °C·min^−1^ to 220 °C, and then increase to 250 °C·min^−1^. Detection of the analyzed compounds was performed using mass spectrometry under the following conditions: Agilent 7895C mass detector chromatograph (Agilent Technology, Palo Alto, CA, USA), ion source temperature: 150 °C; quadrupole temperature: 200 °C; ionization energy: 70 eV. We identified the volatile products based on retention times and by comparing the mass spectra with the NIST 17 library. The maximum coefficient of variation calculated from 3 replicates for each set of samples (REF, FRW, and TTW) was within ±10%.

## 3. Results and Discussion

**Figure 1:**
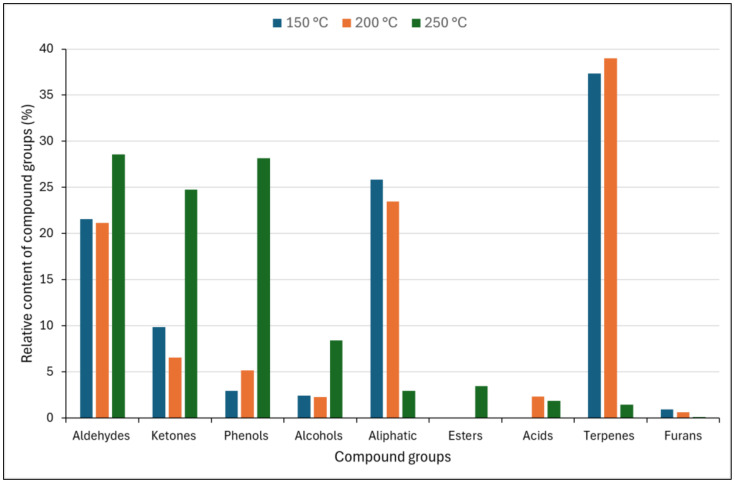
The effect of temperature loading on the relative contents of the identified groups in the reference spruce (REF) wood samples (%).

When subjected to thermal loading, untreated spruce wood releases volatile organic compounds (VOCs) (Table 1). Based on functional groups, the identified VOCs can be divided into nine groups, namely aldehydes (21.13–28.57%), ketones (6.58–24.79%), phenols (2.94%), furans (0.15–0.97%), alcohols (2.27–8.43%), terpenes (1.47–39.01%), aliphatic hydrocarbons (2.96–25.84%), esters (0–3.46%), and acids (0–2.34%) (Figure 1). Aldehydes, ketones, diketones, esters, acids, and furans are the main compounds derived from hemicellulose and cellulose. Conversely, the pyrolysis of lignin primarily results in the production of phenols, phenol, 2-methoxy, phenol, 2,6-dimethoxy, and their derivatives [24]. The effect of temperature loading on the relative contents of these groups is shown in Figure 1.

The majority of gaseous compounds produced during the thermal loading of unmodified spruce wood are carbonyl compounds, aldehydes, and ketones (Table 1, Figure 1). It is worth noting that some authors classify several carbonyl compounds (e.g., furfural) as furans [12,24]. The highest furfural content, formed following the degradation of pentosan, was observed at a temperature of 250 °C (Table 1). Furfural is known to irritate the eyes and skin; however, there is limited evidence that it has carcinogenic effects [25]. Other aldehydes formed from carbohydrates in relatively significant quantities include 5-methyl-2-furancarboxaldehyde, 5-hydroxymethylfurfural, and 5-acetoxymethyl-2-furaldehyde. Other aldehydes (vanillin and 1,2-dimethoxy-4-n-propylbenzene) are also produced following the thermal degradation of lignin. Ketones are produced through the decomposition of carbohydrates and lignin or through subsequent reactions of intermediate products formed during the thermal loading of wood, e.g., 2(5H)-furanone through the oxidation of furfural [26,27].

Phenols are an important group of gaseous products produced during the thermal loading of wood. Creosol, 4-methylguaiacol (an eye and skin irritant), was the most abundant substance found in our experiment. It is a component of wood tar and has been detected as a product of the dry distillation of wood and the combustion of wood pellets [28,29]. Other lignin degradation products, including guaiacol (2-methoxy-phenol) and eugenol, were also found in high concentrations. Among alcohols, furfuryl alcohol (toxic by ingestion and skin contact and moderately toxic by inhalation) occupies an important place.

**Figure 2:**
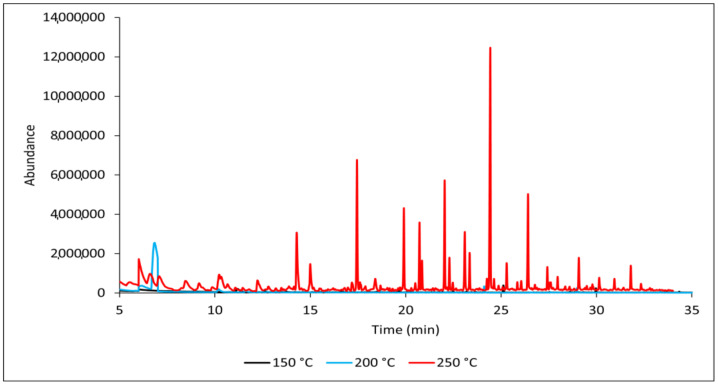
GC-MS chromatograms of reference samples (REF) at different temperatures.

Increasing the thermal loading temperature significantly affects the content of released VOCs, particularly coniferous terpenes, which is consistent with reported data [30,31]. In smaller concentrations, VOCs contain aliphatic hydrocarbons, esters, and acids. These groups of compounds were detected during the burning of wood in other studies [12,32,33]. One of the first compounds released when wood is thermally loaded is acetic acid, which is formed through the degradation of acetylated hemicelluloses and detected in thermally modified hardwoods, oak, and beech [12,34]. Conifer xylans differ from deciduous xylans in that they are not acetylated, and acetic acid is not the main gaseous product in the emission of untreated Norway spruce [35,36]. Under our experimental conditions, acetic acid was not detected. The thermal loading temperature of untreated spruce wood significantly affects the composition and amount of produced VOCs (Figure 2).

To address issues associated with wood flammability, the most common method for improving the fire safety of wood is to modify the wood by impregnating it or surface-coating it with flame retardants [37]. Elemental materials such as phosphorus, boron, silicon, carbon, and metals contribute to fire protection through mechanisms such as thermal insulation, endothermic reactions, oxygen dilution, and char volume expansion. Phosphorus-based compounds promote char formation and inhibit chemical reactions in the gas phase. They catalyze the charring process by promoting the decomposition of both the wood surface and the coating, forming a protective char layer [3,4]. When wood is thermally loaded with a flame-retardant coating, the number and concentration of VOCs are significantly reduced. The main compound produced is furfural; however, other substances, such as terpenes and carbohydrate degradation products, were also identified (Table 2).

**Figure 3:**
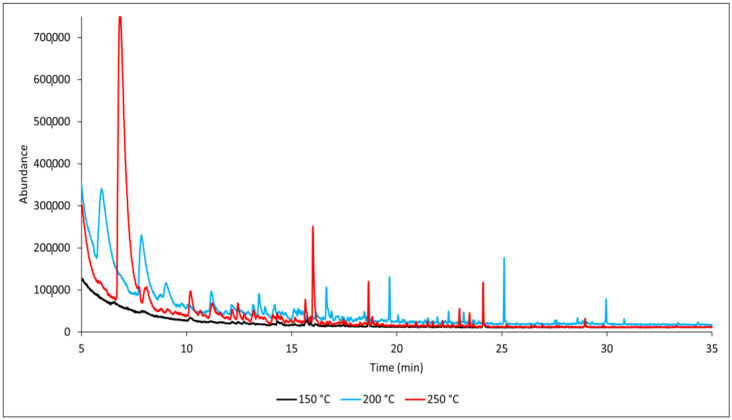
GC-MS chromatograms of flame-retardant-coated samples (FRW) at different temperatures.

Similarly to the reference samples, in the case of samples treated with flame retardant, the thermal loading temperature significantly affects the composition and amount of produced VOCs (Figure 3).

The increasingly widespread use of thermally modified wood necessitates research into the VOCs generated during fire or combustion. A number of authors have demonstrated that the heat treatment of spruce and pine significantly reduces VOC emissions and changes their composition compared to untreated or naturally air-dried wood. Terpene emissions from spruce and pine decrease during the heat treatment process. For both conifers and poplar, heat treatment leads to a reduction in hexanal emissions but causes an increase in furfural emissions for both conifers and broadleaves. Nevertheless, heat treatment represents a suitable method to reduce VOC emissions, thus reducing the potential health risks caused by human exposure to VOCs. Heat treatment of wood makes it an appropriate and non-toxic material for indoor use [25,36]. During heat treatment, wood constituents are degraded, resulting in chemical changes that alter the physical and biological properties of thermally modified wood, including changes in VOC emissions. During thermal treatment, the decomposition of hemicelluloses, in particular, occurs, resulting in a significant decrease in the abundance of VOCs [23,30,35]. In air-dried Norway spruce, terpenes (α–pinene, limonene, and Δ3-carene) were primarily identified. In heat-treated wood (190 °C, 2–3 h), the abundance of terpenes decreased significantly; in addition, furfural, aldehydes, decanal, hexanal, and nonanal were also found in VOCs. Regarding the emissions of heat-treated pine, the most abundant single compounds, furfural, acetic acid, and 2-propanone, accounted for around 60% of the total VOC emissions. None of these compounds were found in the VOC emissions of air-dried wood [30]. In our experiment, only aliphatic hydrocarbons were detected (Table 3), which were also identified together with other compounds in gaseous emissions from residential wood combustion [38]. Evaluation of VOC emissions from Scot’s pine sapwood and heartwood samples after thermal and vacuum–thermal treatment provided confirmation of a decrease in the amount of emitted substances with both heat treatment methods compared to the air-dried samples [39]. Differences in the composition of gaseous products are likely caused by differences in sample preparation, thermal loading, sampling method, and desorption. Based on Hyttinen et al.’s findings [36], some compounds may be formed during analysis in the thermodesorption of adsorption tubes at 250 °C.

**Figure 4:**
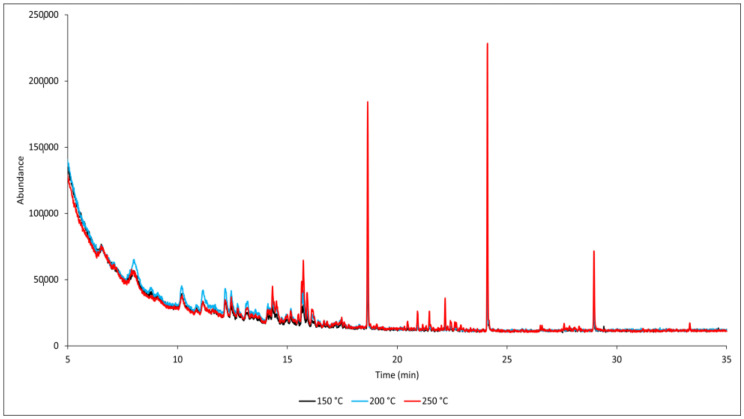
GC-MS chromatograms of thermally treated samples (TTW) at different temperatures.

Similarly to the reference and flame-retardant-treated samples, a temperature of 250 °C dramatically increases the concentration of VOCs produced (Figure 4).

**Figure 5:**
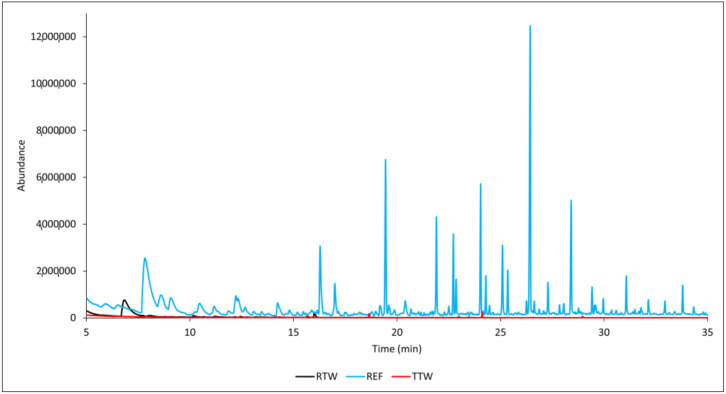
GC-MS chromatograms of samples subjected to different treatment methods at 250 °C.

The significant difference in the number of compounds identified from the FRW samples compared to the reference samples (REF) was due to the use of a retardant. By employing this treatment method, the greatest abundance of volatile products was identified in the untreated wood samples and the lowest abundance was identified in the heat-treated wood samples (TTW) (Figure 5).

## 4. Conclusions

The thermal loading of wood, common in various woodworking processes and techniques, results in the production of a range of flammable and non-flammable gases and toxic compounds. Their amount is influenced by the wood treatment strategy and the conditions of thermal loading (e.g., wood type, temperature, and environment). In this study, we monitored the gaseous products produced during the thermal loading of untreated spruce wood (REF), thermally treated wood (TTW), and wood treated with a flame retardant (FRW) at temperatures of 150 °C, 200 °C, and 250 °C. In the untreated wood samples, the number and concentration of gaseous products increased with the thermal treatment temperature, with the most abundant being carbonyl compounds and phenols formed through the decomposition of lignin, cellulose, and hemicelluloses. In the gaseous products of wood treated with a flame retardant, the number of compounds present was greatly reduced. The main compound was furfural, demonstrating partial degradation of hemicelluloses. A further decrease in the quantity of gaseous products was observed during the thermal treatment of thermally modified wood, with only aliphatic hydrocarbons being present. For all types of samples, the number of compounds increased sharply up to a temperature of 250 °C, which can impact the combustion process and simultaneously endanger human health through exposure to toxic substances. The challenge for the authors of future studies is to find suitable wood treatment methods and flame retardants that do not produce toxic substances while maintaining the temperature of the wood during fire below 200 °C.

## Figures and Tables

**Table 1:** Compounds and their contents in VOCs released from reference (REF) wood subjected to thermal loading at different temperatures (peak area ×10^5^).

Group	Compound	RT (min)	Temperature (°C)		
			150	200	250
Aldehydes	Vanillin	24.097	10.1	13.2	52.7
	Furfural	6.841	0.0	0.0	306.7
	2-Furancarboxaldehyde, 5-methyl-	11.230	0.0	0.0	53.1
	5-Hydroxymethylfurfural	19.406	0.3	0.3	20.7
	5-Acetoxymethyl- 2-furaldehyde	21.724	0.0	0.0	44.3
	4-Hydroxy- 2-methoxycinnamaldehyde	31.938	0.3	0.0	18.7
	5-Norbornane- 2-carboxaldehyde	12.783	0.0	0.0	11.4
	1,2-Dimethoxy- 4-n-propylbenzene	26.847	0.0	0.0	10.5
Ketones	2(5H)-Furanone	9.472	0.0	0.0	79.9
	4-Methyl-5H-furan-2-one	11.662	0.0	0.0	31.4
	2-Furanone, 2,5-dihydro-3,5-dimethyl	12.460	1.5	1.1	5.1
	4-Methyl-5H-furan-2-one	13.797	0.0	0.0	18.6
	2(3H)-Furanone, 5-acetyldihydro-	16.483	0.6	0.4	2.7
	4-Methoxycarbonyl- 4-butanolide	19.675	0.0	0.0	6.1
	1-(acetyloxy)-2-Propanone (Acetoxyacetone)	8.081	0.0	0.0	150.5
	2-Butanone, 4-(acetyloxy)-	12.083	0.0	0.0	12.2
	1,2-Cyclopentanedione, 3-methyl-	13.236	1.4	1.4	28.9
	Levoglucosenone	15.997	1.0	0.9	37.7
	2-Propanone, 1-(4-hydroxy- 3-methoxyphenyl)-	27.408	0.0	0.0	73.8
Phenols	Phenol, 2-methyl-	14.196		0.0	5.3
	Phenol, 2-methoxy-	15.286	0.0	1.3	74.8
	Creosol	18.446	0.0	0.0	108.5
	Phenol, 4-ethyl-2-methoxy-	20.905	0.7	0.6	60.3
	2-Methoxy-4-vinylphenol	21.854	0.0	0.0	42.3
	Eugenol	23.040	0.4	0.4	73.5
	Phenol, 2-methoxy-4-propyl-	23.288	0.0	0.0	21.2
	trans-Isoeugenol	24.345	0.0	0.3	25.2
	Phenol, 2-methoxy-4-propyl-	25.629	0.0	0.0	21.2
	(Z)-4-(But-1-en-1-yl)guaiacol	26.082	0.0	0.0	4.2
	Apocynin	26.286	0.0	0.0	38.3
	(E)-4-(But-1-en-1-yl)guaiacol	27.764	0.3	0.7	4.3
	4-(1-Hydroxyallyl)- 2-methoxyphenol	28.422	0.0	0.0	31.7
	Phenol, 2-methoxy- 4-(1-propenyl)-, Acetate	29.360	0.0	0.0	5.4
Alcohols	3-Pyridinol	14.488	1.2	0.8	7.1
	Orcinol	14.887	0.0	0.3	11.2
	Benzenepropanol, 4-hydroxy-3-methoxy-	30.072	0.0	0.3	1.6
	2-furanmethanol (Furfuryl alcohol)	7.617	0.0	0.0	132.9
Aliphatic	Undecane	15.652	5.2	3.6	4.9
	Heptane, 4-ethyl-	18.607	0.0	0.3	19.4
	Tridecane	21.465	1.4	1.4	0.0
	Hexadecane	28.961	5.0	7.8	15.6
	Heptadecane	33.329	0.8	1.5	7.4
	Oktadecane	37.126	0.4	0.4	6.4
Esters	Benzoic acid, 4-hydroxy- 3-methoxy-, methyl ester	27.052	0.0	0.0	15.4
	Benzeneacetic acid, 4-hydroxy-3-methoxy-, methyl ester	28.594	0.0	0.0	5.5
	Ethyl-.beta.-(4-hydroxy-3-methoxy-phenyl)propionate	32.790	0.0	0.0	18.0
	7-Oxodehydroabietic acid, methyl ester	42.475	0.0	0.0	15.7
	1-Phenanthrenecarboxylic acid,1,2,3,4,4a,10a-hexahydro-1,4a-dimethyl-7-(1-methyl ethyl)-,methyl ester, [1R-(1.alpha.,4a.beta.,10a.alpha.)]-	40.534	0.0	0.0	8.1
Terpenes	α-Pinene	10.173	13.0	18.8	26.6
	β-Pinene	11.597	2.5	6.0	0.0
	Δ3-carene	12.729	3.1	0.0	0.0
Acids	Phenylacetylformic acid, 4-hydroxy-3-methoxy-	31.140	0.0	0.0	10.6
	Methyl dehydroabietate	40.577	0.0	1.5	23.0
Furans	Furane, 2,5-dihydro-2,5-dimethoxy-	20.312	0.5	0.4	2.7

**Table 2:** Compounds and their contents in VOCs released from flame-retardant-treated (FRW) wood subjected to thermal loading at different temperatures (peak area ×10^5^).

Group	Compound	RT (min)	Temperature (°C)		
			150	200	250
Aldehydes	Furfural	6.827	0.00	31.04	391.10
Ketones	Levoglucosenone	16.016	0.37	0.99	5.86
	4-Hexen-3-one	23.504	0.00	0.26	6.86
Terpenes	α-pinene	10.184	0.00	5.27	9.68
Aliphatic	Undecane	15.649	0.43	2.41	3.81
	Dodecane	18.658	1.52	3.67	3.99
	4-Methyl-2-hexene, c&t	22.986	0.00	0.42	7.05
	Tetradecane	24.105	1.96	13.95	6.35
	Hexadecane	28.958	0.89	12.13	2.07

**Table 3:** Compounds and their contents in VOCs released from thermally treated (TTW) wood subjected to thermal loading at different temperatures (peak area ×10^5^).

Group	Compound	RT (min)	Temperature (°C)		
			150	200	250
Aliphatic	Dodecane	18.658	1.6	1.7	3.8
	Tetradecane	24.105	1.8	1.6	5.3
	Hexadecane	28.958	0.6	0.6	1.4

## Data Availability

The original contributions presented in this study are included in the article; further inquiries can be directed to the corresponding author.

## References

[1] Lowden L.A., Hull T.R. (2013). Flammability Behaviour of Wood and a Review of the Methods for Its Reduction. Fire Sci. Rev..

[2] Mai C., Zhang K., Niemz P., Teischinger A., Sandberg D. (2023). Wood Chemistry. Springer Handbook of Wood Science and Technology.

[3] Mensah R.A., Jiang L., Renner J.S., Xu Q. (2023). Characterisation of the Fire Behaviour of Wood: From Pyrolysis to Fire Retardant Mechanisms. J. Therm. Anal. Calorim..

[4] Lee Y.X., Wang W., Lei Y., Xu L., Agarwal V., Wang C., Yeoh G.H. (2025). Flame-Retardant Coatings for Wooden Structures. Prog. Org. Coat..

[5] Reinprecht L. (2016). Wood Deterioration, Protection and Maintenance.

[6] Júda M., Sydor M., Rogoziński T., Kučerka M., Pędzik M., Kminiak R. (2023). Effect of Low-Thermal Treatment on the Particle Size Distribution in Wood Dust after Milling. Polymers.

[7] Gaff M., Kačík F., Gašparík M., Todaro L., Jones D., Corleto R., Makovická Osvaldová L., Čekovská H. (2019). The Effect of Synthetic and Natural Fire-Retardants on Burning and Chemical Characteristics of Thermally Modified Teak (*Tectona Grandis* L. f.) Wood. Constr. Build. Mater..

[8] Li T., Wu Q., Lu W., Zhang J., Yue Z., Jie Y., Zhang J., Cheng Z., Ji W., Wu J. (2024). Effects of Different Accelerated Aging Modes on the Mechanical Properties, Color and Microstructure of Wood. J. Build. Eng..

[9] Sandberg D., Kutnar A., Karlsson O., Jones D. (2021). Wood Modification Technologies: Principles, Sustainability, and the Need for Innovation.

[10] Candelier K., Chaouch M., Dumarçay S., Pétrissans A., Pétrissans M., Gérardin P. (2011). Utilization of Thermodesorption Coupled to GC–MS to Study Stability of Different Wood Species to Thermodegradation. J. Anal. Appl. Pyrol..

[11] Candelier K., Dumarçay S., Pétrissans A., Pétrissans M., Kamdem P., Gérardin P. (2013). Thermodesorption Coupled to GC–MS to Characterize Volatiles Formation Kinetic during Wood Thermodegradation. J. Anal. Appl. Pyrol..

[12] Xu J., Zhang Y., Shen Y., Li C., Wang Y., Ma Z., Sun W. (2019). New Perspective on Wood Thermal Modification: Relevance between the Evolution of Chemical Structure and Physical-Mechanical Properties, and Online Analysis of Release of VOCs. Polymers.

[13] Poletto M., Zattera A.J., Santana R.M.C. (2012). Thermal Decomposition of Wood: Kinetics and Degradation Mechanisms. Bioresour. Technol..

[14] Vargun E., Baysal E., Turkoglu T., Yuksel M., Toker H. (2019). Thermal Degradation of Oriental Beech Wood Impregnated with Different Inorganic Salts. Maderas Cienc. Tecnol..

[15] Dietenberger M., Hasburgh L. (2016). Wood Products Thermal Degradation and Fire. Ref. Modul. Mater. Sci. Mater. Eng..

[16] Gaff M., Kačík F., Sandberg D., Babiak M., Turčani M., Niemz P., Hanzlík P. (2019). The Effect of Chemical Changes during Thermal Modification of European Oak and Norway Spruce on Elasticity Properties. Compos. Struct..

[17] Ornaghi H.L., Ornaghi F.G., Motta Neves R., Magalhães De Oliveira D., Poletto M. (2021). Thermal Decomposition of Wood Fibres: Thermal Simulation Using the F-Test Statistical Tool. Cellul. Chem. Technol..

[18] Albert C.M., Liew K.C. (2024). Recent Development and Challenges in Enhancing Fire Performance on Wood and Wood-Based Composites: A 10-Year Review from 2012 to 2021. J. Bioresour. Bioprod..

[19] De Angelis M., Humar M., Kržišnik D., Tamantini S., Romagnoli M. (2022). Influence of Thermal Modification and Impregnation with Biocides on Physical Properties of Italian Stone Pine Wood (*Pinus Pinea* L.). Appl. Sci..

[20] Popescu C.-M., Pfriem A. (2020). Treatments and Modification to Improve the Reaction to Fire of Wood and Wood Based Products—An Overview. Fire Mater..

[21] Lazar S.T., Kolibaba T.J., Grunlan J.C. (2020). Flame-Retardant Surface Treatments. Nat. Rev. Mater..

[22] Lai Y., Liu X., Davies M., Fisk C., Holliday M., King D., Zhang Y., Willmott J. (2024). Characterisation of Wood Combustion and Emission under Varying Moisture Contents Using Multiple Imaging Techniques. Fuel.

[23] Pohleven J., Burnard M.D., Kutnar A. (2019). Volatile Organic Compounds Emitted from Untreated and Thermally Modified Wood—A Review. Wood Fiber Sci..

[24] Sokamte Tegang A., Mbougueng P.D., Sachindra N.M., Douanla Nodem N.F., Tatsadjieu Ngoune L. (2020). Characterization of Volatile Compounds of Liquid Smoke Flavourings from Some Tropical Hardwoods. Sci. Afr..

[25] Adamová T., Hradecký J., Pánek M. (2020). Volatile Organic Compounds (VOCs) from Wood and Wood-Based Panels: Methods for Evaluation, Potential Health Risks, and Mitigation. Polymers.

[26] Tuppurainen V., Fleitmann L., Kangas J., Leonhard K., Tanskanen J. (2024). Conceptual Design of Furfural Extraction, Oxidative Upgrading and Product Recovery: COSMO-RS-Based Process-Level Solvent Screening. Comput. Chem. Eng..

[27] Palai Y.N., Fukuoka A., Shrotri A. (2024). Unlocking the Potential of 5-Hydroxy-2(5H)-Furanone as a Platform for Bio-Based Four Carbon Chemicals. ACS Catal..

[28] Zhang Y., Yang M., Yuan D., Li C., Ma Y., Wang S., Wang S. (2023). Fire Hazards of PMMA-Based Composites Combined with Expandable Graphite and Multi-Walled Carbon Nanotubes: A Comprehensive Study. Fire Saf. J..

[29] Mizera K., Borucka M., Przybysz J., Gajek A. (2024). Identification of Substances Emitted During Combustion and Thermal Decomposition of Wood-Based Materials. Chem. Eng. Trans..

[30] Manninen A.-M., Pasanen P., Holopainen J.K. (2002). Comparing the VOC Emissions between Air-Dried and Heat-Treated Scots Pine Wood. Atmos. Environ..

[31] Kačík F., Veľková V., Šmíra P., Nasswettrová A., Kačíková D., Reinprecht L. (2012). Release of Terpenes from Fir Wood during Its Long-Term Use and in Thermal Treatment. Molecules.

[32] Atiku F.A., Lea-Langton A.R., Bartle K.D., Jones J.M., Williams A., Burns I., Humphries G. (2017). Some Aspects of the Mechanism of Formation of Smoke from the Combustion of Wood. Energy Fuels.

[33] Adamová T., Hradecký J., Prajer M. (2019). VOC Emissions from Spruce Strands and Hemp Shive: In Search for a Low Emission Raw Material for Bio-Based Construction Materials. Materials.

[34] Shen D.K., Gu S., Bridgwater A.V. (2010). Study on the Pyrolytic Behaviour of Xylan-Based Hemicellulose Using TG–FTIR and Py–GC–FTIR. J. Anal. Appl. Pyrol..

[35] Peters J., Fischer K., Fischer S. (2008). Characterization of Emissions from Thermally Modified Wood and Their Reduction by Chemical Treatment. BioResources.

[36] Hyttinen M., Masalin-Weijo M., Kalliokoski P., Pasanen P. (2010). Comparison of VOC Emissions between Air-Dried and Heat-Treated Norway Spruce (*Picea Abies*), Scots Pine (*Pinus Sylvesteris*) and European Aspen (*Populus Tremula*) Wood. Atmos. Environ..

[37] Liang Y., Jian H., Deng C., Xu J., Liu Y., Park H., Wen M., Sun Y. (2023). Research and Application of Biomass-Based Wood Flame Retardants: A Review. Polymers.

[38] McDonald J.D., Zielinska B., Fujita E.M., Sagebiel J.C., Chow J.C., Watson J.G. (2000). Fine Particle and Gaseous Emission Rates from Residential Wood Combustion. Environ. Sci. Technol..

[39] Sivrikaya H., Tesařová D., Jeřábková E., Can A. (2019). Color Change and Emission of Volatile Organic Compounds from Scots Pine Exposed to Heat and Vacuum-Heat Treatment. J. Build. Eng..

